# Development of ultra‐sensitive and reproducible measurement system for diagnosis of Alzheimer's disease

**DOI:** 10.1002/alz70856_102483

**Published:** 2025-12-25

**Authors:** Keisuke Shimizu, Kenta Tosaka, Noboru Ogihara, Taichi Matsunaga, Hiroshi Satou

**Affiliations:** ^1^ TOSOH Corporation, Ayase‐shi, Kanagawa, Japan

## Abstract

**Background:**

Alzheimer's disease (AD) have become more prevalent in aging societies. Then, plasma biomarker to assess of AD‐related pathological stages is required. However, such AD‐related biomarkers in blood exist at extremely low levels and are difficult to detect precisely and reproducibly.

**Method:**

The measurement system was constructed by using brain natriuretic peptide(BNP) as a model biomarker. The outline of the system is as follows. (1) BNP in sample is captured by anti‐BNP antibody modified magnetic beads followed by enzyme labeling. (2) Magnetic beads with capturing BNP are enclosed in a microwell array for ultra‐sensitive detection of the marker. In our method, multiple beads are enclosed in a microwell to measure as many beads as possible and improve measurement reproducibility. (3) BNP is quantified by integrating the fluorescence intensity which generated by the enzymatic reaction in each microwells. BNP standards were measured in triplicate by developed system and calculated the limit of detection (LOD). The measurement system for an AD marker was also established similarly.

**Result:**

The LOD of BNP was 2.5 fM (8.6 fg/mL), which was significantly sensitive compared to the conventional immunoassay system. Furthermore, the coefficient of variation (CV) of 7.2 fM (25 fg/mL) BNP was 8.1%, so that reproducibility in the low concentration range was also satisfactory. An AD marker was also detected by using the system.

**Conclusion:**

We developed ultra‐sensitive and reproducible measurement system and it can be applied to the detection of AD‐related biomarkers in blood.

